# Pan genome and CRISPR analyses of the bacterial fish pathogen *Moritella viscosa*

**DOI:** 10.1186/s12864-017-3693-7

**Published:** 2017-04-20

**Authors:** Christian Karlsen, Erik Hjerde, Terje Klemetsen, Nils Peder Willassen

**Affiliations:** 1 Department of Food Safety and Infection Biology, Norwegian University of Life Sciences (NMBU), Pb 8146 Dep., 0033 Oslo, Norway; 2 Present address: Nofima AS, Division of Aquaculture, PO Box 210, Ås, N-1431 Norway; 3 Department of Chemistry, Faculty of Science and Technology, University of Tromsø, N-9037 Tromsø, Norway; 4 The Norwegian Structural Biology Centre, University of Tromsø, N-9037 Tromsø, Norway

**Keywords:** *Moritella viscosa*, CRISPR-Cas, Mobile genetic element, Atlantic salmon pathogen

## Abstract

**Background:**

Winter-ulcer *Moritella viscosa* infections continue to be a significant burden in Atlantic salmon (*Salmo salar* L.) farming. *M. viscosa* comprises two main clusters that differ in genetic variation and phenotypes including virulence. Horizontal gene transfer through acquisition and loss of mobile genetic elements (MGEs) is a major driving force of bacterial diversification. To gain insight into genomic traits that could affect sublineage evolution within this bacterium we examined the genome sequences of twelve *M. viscosa* strains. Matches between *M. viscosa* clustered, regularly interspaced, short palindromic, repeats and associated *cas* genes (CRISPR-Cas) were analysed to correlate CRISPR-Cas with adaptive immunity against MGEs.

**Results:**

The comparative genomic analysis of *M. viscosa* isolates from across the North Atlantic region and from different fish species support delineation of *M. viscosa* into four phylogenetic lineages. The results showed that *M. viscosa* carries two distinct variants of the CRISPR-Cas subtype I-F systems and that CRISPR features follow the phylogenetic lineages. A subset of the spacer content match prophage and plasmid genes dispersed among the *M. viscosa* strains. Further analysis revealed that prophage and plasmid-like element distribution were reflected in the content of the CRISPR-spacer profiles.

**Conclusions:**

Our data suggests that CRISPR-Cas mediated interactions with MGEs impact genome properties among *M. viscosa*, and that patterns in spacer and MGE distributions are linked to strain relationships.

**Electronic supplementary material:**

The online version of this article (doi:10.1186/s12864-017-3693-7) contains supplementary material, which is available to authorized users.

## Background

The genus *Moritella* comprises seven psychrophilic species associated with deep seawater and ocean sediments. *Moritella viscosa* is the only species so far associated with fish pathogenicity, being the causative agent of winter-ulcer disease in farmed salmonids [1, 2]. Outbreaks occur in salmonid aquaculture across the North Atlantic [3–7] and infected fish develop chronic skin ulcers that may be followed by terminal septicaemia [3, 6]. Two major phenotypic and genotypic clades (‘typical’ and ‘variant’) have been identified in *M. viscosa* [5]. It is suggested that phylogenetic lineages within *M. viscosa* have evolved compatibility factors that adapt typical *M. viscosa* to host-specific virulence [8].

Phenotypic and genotypic variations may originate from horizontal gene transfer (HGT) that introduces new elements through mechanisms such as conjugation, transformation and transduction through bacteriophage-mediated DNA or plasmid transfer [9]. Acquisition or loss of mobile genetic elements (MGEs) could alter virulence properties, e.g. by introducing a novel toxin or surface alteration in a strain [9]. Bacteriophage might also present a danger to the host bacteria as bacteriophages can cause bacteriolysis [10]. Temperate bacteriophages have, unlike virulent phages, the ability to integrate their DNA into the bacterium’s chromosome where it enters a prophage dormant state replicating along with the host genome.

In response, bacteria have mechanisms to resist infection of MGEs. One is the clustered, regularly interspaced, short palindromic repeats (CRISPRs) flanked by CRISPR-associated (*cas*) genes. The CRISPR-Cas system is used in most archaea [11] and are widespread across diverse bacteria [12, 13] including the phylum Cyanobacteria [14]. The system can act against invading foreign viruses and plasmids by targeting DNA in a sequence specific manner [15]. CRISPRs consist of short (23–47 bp) highly conserved repeats separated by variable sequences called spacers. Spacers are acquired mostly independently from foreign DNA, and only a smaller subset is transmitted vertically [15]. The Cas proteins are involved in this defence mechanism, both processing, binding and targeting of foreign DNA, and integrating novel spacer units into the CRISPR locus [15]. The complimentary sequence to spacers that originate from invading genetic elements are termed protospacers. Spacers incorporated into the CRISPR loci are transcribed acting as guides that anneal to the complementary protospacers of the invading genetic element. The CRISPR-Cas mechanism will then degrade the foreign nucleic acids. The invader can in turn evade this resistance by modifying the targeted DNA sequence generating CRISPR escape mutations [16]. Thus, CRISPRs are considered to be a form of acquired immunity from past infections which may provide insights into bacterial niche adaptation, evolution and phage-host dynamics that have occurred within the bacterial populations [17]. CRISPR is rapidly evolving in the genomes of some microbial pathogens and can be used to detect and genotype clinical isolates of *Mycobacterium tuberculosis* [18], *Corynebacterium diphtheria* [19] and *Salmonella enterica* subs. *enterica* [20]. However, CRISPR distribution may not always correlate to phylogenetic relationships, as independent evolution in select lineages can advance in part by HGT and environmental differences in phage predation [13].

In this study, a bioinformatics approach has been used to resolve genomic diversity between twelve *M. viscosa* isolated from different geographical locations and fish species. We analysed the CRISPR-Cas systems and the CRISPR locus organization to determine relatedness to strain origin. All *M. viscosa* spacers were then examined to establish spacer diversity and to identify the protospacers of targeted genes. In order to examine the potential function of the CRISPR-Cas system in *M. viscosa* all spacers were searched against the twelve *M. viscosa* genomes, and examined by relating the results obtained to MGE distribution in the corresponding strains. Our analyses suggest that the CRISPR-Cas system in *M. viscosa* is an important determinant of genetic transfer involved in prophage and plasmid distribution influencing the evolution of this fish pathogenic species.

## Methods

### Bacterial strains and DNA extraction

The 12 *M. viscosa* strains analysed here include representatives isolated from different fish species that span the geographical area of occurring outbreaks of winter-ulcer disease across the North Atlantic region (Additional file 1: Table S1). The isolates include both typical and variant *M. viscosa*, which were categorized as per standard biochemical and phenotypic methods as well as sequence analysis [2, 5, 8, 21]. The complete genome of the virulent *M. viscosa* MV 0609139 [22, 23] was used as reference. Strains were cultured in Luria-Bertani broth containing 3.5% NaCl at 12 °C. DNA was extracted using the Qiagen DNeasy blood and tissue kit protocol for Gram-negative bacteria.

### Genome sequencing, assembly and annotation

Sequencing libraries for the bacterial isolates were made using the Nextera XT kit according to the manufacturer’s protocol, and the fragment size distribution analysed to be 500–1000 bp using the Agilent 2100 Bioanalyzer System. The sample libraries were multiplexed and sequenced in a single run on a MiSeq machine (Illumina) using v3 reagents with 2 × 150 cycles according to the manufacturer’s instructions. This yielded an average of 2.06 million reads per bacterial isolate. The twelve genomes were assembled *de novo* using CLC Genomics Workbench v6.5 (https://www.qiagenbioinformatics.com/) with default parameters, not performing scaffolding and with 500 bases as minimum cutoff length for each contig. The resulting contigs were mapped against our reference genome using standard Nucmer settings with ABACAS v1.3.1 [24]. Unmapped contigs were included by appending them to the output fasta-file with the mapped contigs. This was followed by concatenation using the six-frame stop-codon "CTAGCTAGCTAG" as separators between contigs. Glimmer v3.02 [25] was then used to identify possible protein coding genes (CDSs) on the concatenated sequences before subsequent annotation by basic local alignment search tool (BLAST), using protein-protein BLASTp (UniProt database release 01 2014) [26, 27], HMMER3 v3.1b1 (hmmscan applying Pfam database v27.0) [28, 29] and SignalP v4.0 [30]. Genome sequences are available from European Nucleotide Archive (ENA) through the study accession number PRJEB1601. Accession number for each genome is listed in Additional file 2: Table S2A.

### Orthologue identification

Clustering of orthologous genes was done by OrthoMCL v1.4 [31], with the input consisting of 12 multifasta-files containing the predicted CDSs from each sequenced strains. The parameters were set at 90 percent identity cutoff and 20 percent match cutoff for the clustering algorithm. BLAST p-value cutoff, max weight and MCL inflation were set to default.

### Pan genome analysis

A pan genome of all 12 strains was identified using the 4720 clusters determined by OrthoMCL. This was achieved by extracting each cluster separately before creating a precursory consensus sequence from each cluster using the script Consensus.pl available on Github (https://github.com/josephhughes/Sequence-manipulation). All consensus sequences were then amassed in a single multifasta-file in the same order as the orthoMCL output while appending the 967 unclustered (unique) genes. For the sake of clarity, cluster information, consensus sequence lengths and annotation were additionally handled in an excel spreadsheet to sort the number of genes in each cluster, from highest to lowest with the associated strains. Gene clusters present in all strains were defined as being part of the ‘core’ genome. Gene clusters present in all strains containing additional paralogs were defined as ‘core plus’, while clusters not represented by all strains were part of the ‘accessory’ genome. Genes only present in single strains were defined as ‘unique’. The ordered data was used to generate a pan genome diagram using Circos [32].

### Gene ontology

Annotation of Gene Ontology (GO) [33] was also performed on the predicted CDSs using InterProScan [34]. The resulting outputs were counted using the web tool WEGO [35], where GO data from the four uppermost levels of the ontologies were collected for each strain and compared in a line plot.

### Whole genome phylogenetics

Single-nucleotide polymorphisms (SNPs) were identified and a Maximum likelihood tree reconstructing the phylogenetic relationship between the isolates was performed on the core genome using the alignment free software kSNP [36]. A gene content tree was constructed from a binary pan genome cluster matrix (presence or absence of genes in each isolate relative to the other isolates) generated with GET_HOMOLOGUES [37] using the discrete character parsimony algorithm. The tree comparison was performed with EPoS [38] with ten tanglegram computations.

### Prophage prediction

Prophages in *M. viscosa* genomes were identified using the Phage Search Tool (PHAST) webserver [39]. We further checked whether the *M. viscosa* phylogeny was linked to presence of certain prophages.

### CRISPR-Cas analysis and protospacer identification

The orthologue analysis identified CRISPR related Cas genes in variant *M. viscosa*, and the genomes of all *M. viscosa* were searched for CRISPR arrays using CRISPRfinder [40] and by BLAST searches of the identified *cas* genes in variant *M. viscosa* against a local database generated from the CDSs of all *M. viscosa* genomes in BioEdit [41]. Cas gene sequences and the deduced amino acid sequences from these genes within *M. viscosa* CRISPR type I and CRISPR type II were aligned using ClustalW. To examine the potential significance of the CRISPR-Cas system in *M. viscosa,* all *M. viscosa* spacers (Additional file 3) were searched against CRISPRTarget [42] to identify possible protospacers. A match against the GenBank-Phage or RefSeq-Plasmid databases was counted when a spacer had ≤4 SNPs over the length of 32 nucleotides. A relative measure of relatedness was calculated from BLASTn results generated from pairwise comparison of each spacers to all *M. viscosa* spacers. Spacers from one strain that matched to the spacer-array of another *M. viscosa* were defined as ≤1 SNP (31/32 nucleotides). All *M. viscosa* spacers were further utilized in BLASTn searches against the CDSs of all *M. viscosa* to identify possible protospacers or targeted genes within *M. viscosa*. The investigated *M. viscosa* strains were found to carry a range of different MGEs and the detection of protospacers were further related to the MGE distribution in the corresponding strains. The putative uncharacterized protein encoded by K56_4594 and MT2528_4809 in plasmid B was analysed further by utilizing the Phyre2 web portal for protein modelling, prediction and analysis [43].

## Results

### General features and comparisons and the core genome of *M. viscosa*

**Fig. 1:**
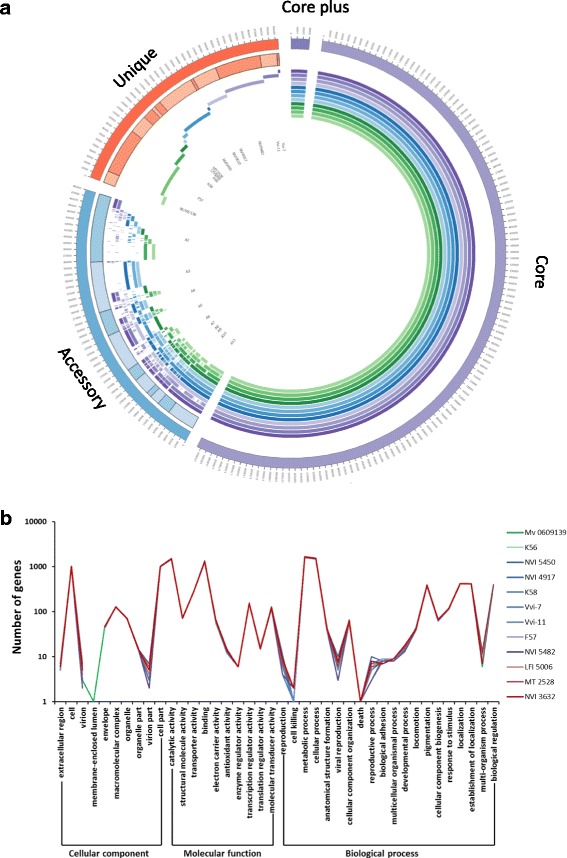
Comparative genome content of twelve *Moritella viscosa*. **a** The outermost circle indicates the classification into core plus, core, accessory and unique genes in the pan genome of *M. viscosa*. Internal circles indicate gene presence (solid colour) or absence (unfilled) of each gene in each of the 12 strains examined. Genes are represented only once in the diagram, but the gene order in the different core, accessory and unique segments are discontinuous, since genes may be represented in different segments. Circles from outer to inner are representing strains in the following order. Purple; Vvi-7, Vvi-11, NVI 5482, NVI 4917. Blue; NVI 3632, NVI 5450, MT 2528, LFI 5006. Green; K58, K56, F57, MV 0609139. **b** Gene ontology (GO) term category distribution. Functional classification of genes with GO terms encoded by *M. viscosa* genomes is displayed for each *M. viscosa* isolate as a line chart plotted against a logarithmic scale. The number of genes represents the amount annotated into the corresponding term of three GO categories at level 2. The twelve *M. viscosa* isolates are indicated by colour codes

The comparative genome content of twelve *M. viscosa* is shown in Fig. 1. The completeness of the draft genomes were assessed by mapping onto the complete reference genome of *M. viscosa* MV 0609139 [22]. Percentage of bases mapped to the reference genome range from 61.7 to 94.8% with an average of 84.0%. Genome sizes and number of predicted genes ranged from 4.96 to 5.3 Mbp and 4532 to 4924, respectively. General genomic and sequence statistics and the numbers of CDSs shared between or being unique to *M. viscosa* strains are shown in Additional file 2: Table S2A-D. The average number of genes was 4718, with 3737 core genes found in all strains. Orthologue analysis (Fig. 1) revealed that strains share between 465 and 1028 dispensable (accessory) genes and that number of strain specific (unique) genes (in total 1888) varied between 22 to 362 genes in each strain. Grouping all functional genes from the twelve *M. viscosa* genomes identified 5589 pan genomic gene clusters. Comparing the core genes to the pan genome cluster showed that the core genome accounts for 67% of the pan genome.

### Functional categories of predicted *M. viscosa* genes

Identified genes were categorized by GO assignments into 40 functional processes within the “cellular component”, “molecular function” and “biological process” categories at level 2 (Fig. 1b). The homology assignments revealed little discrepancy in the distribution of genes within the *M. viscosa* genomes investigated. Refining the categorization further (results not shown) revealed that in the cellular component category the largest numbers of genes grouped into the sub-category membrane or membrane part. For genes within molecular function, the largest sub-categories were nucleic acid binding, transferase and hydrolase activity. In the biological process category, genes sub-grouped into cellular-, primary-, nitrogen-, and biosynthetic-metabolic processes. The sub-categorization of the “biological process” category revealed further that most discrepancies are associated with MGEs such as prophage-associated genes.

### Relationships among the *M. viscosa* genomes

**Fig. 2:**
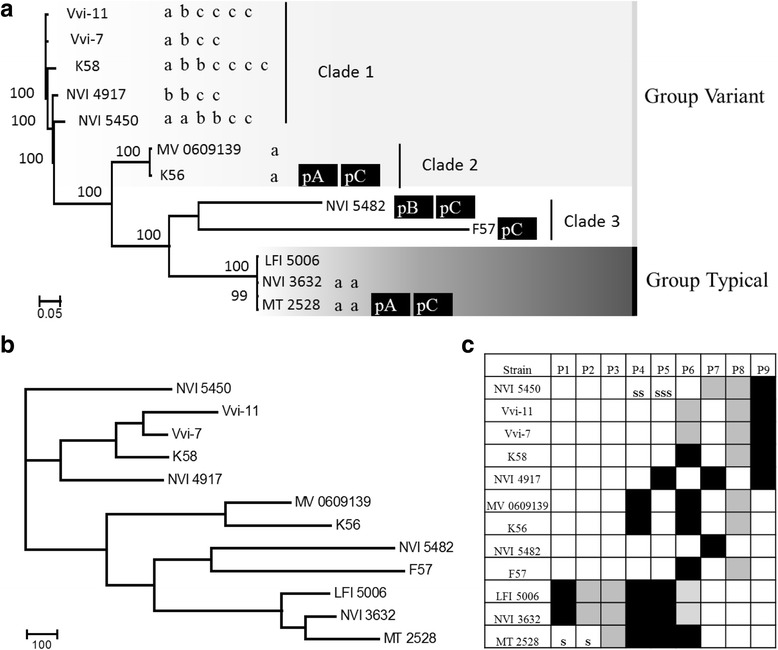
SNPs phylogeny, protospacers, gene content tree and predicted mobile genetic element distribution. **a** The phylogenetic tree generated from the core genome SNPs of the twelve *Moritella viscosa* isolates using the Maximum likelihood method. Bootstrap values of 1000 repetitions are shown adjacent to nodes. The twelve *M. viscosa* strains used in this study separates into four different lineages; variant *M. viscosa* (as defined by [5]) sub-grouped into clade 1–3, and typical (as defined by [5]) *M. viscosa*. Colour-coding of the phylogenetic tree represent the presence of CRISPR-Cas system type I (light grey, clade 1 and 2), and CRISPR-Cas system type II (dark grey, typical *M. viscosa*). Variant clade 3 without colour has no predicted CRISPR-Cas system. Spacer distribution that matches plasmid-like elements are show for each isolate and denoted to the right of strains as a = spacer matching plasmid A, b = spacer matching plasmid B, and c = spacer matching plasmid C. The following black box represent the presence of pA = plasmid A, pB = plasmid B or pC = plasmid C. **b** Gene content tree of the twelve *M. viscosa* genomes examined. The topology of the tree is congruent, with exception of the NVI 5450 strain, to the SNP phylogeny providing further support to the relationships among the divergent *M. viscosa* lineages. **c** Distribution of the putative prophages in *M. viscosa*. The strain organization in the table column reflects the phylogenetic relationships in the gene content tree and the SNP tree. There are nine prophages that are shared by more than one genome. Cells highlighted in black or grey are categorized by the PHAST prediction tool as an intact or questionable prophage region, respectively. The letter(s) S-SSS denote the number of matching spacers to this prophage

*M. viscosa* can be separated into two major phenotypically and genetically different clusters (typical and variant) by haemolytic activity, which is consistent with Western blot, plasmid profile, pulsed field gel electrophoresis and *gyrB* gene sequence analyses [5]. However, the whole-genome phylogenetic SNP analysis and the gene content tree (Fig. 2) do not separate strains into the present typical/variant classification. Strains LFI 5006, NVI 3632 and MT 2528 from Norwegian and Scottish Atlantic salmon do group into typical *M. viscosa* as previously described [5, 8]. The variant *M. viscosa* are sublineaged into three clades where both clade 2 and clade 3 form a cluster with typical *M. viscosa*. Clade 2 contains isolates from Norwegian (strain MV 0609139) and Icelandic (strain K56) farmed Atlantic salmon. Clade 3 contains isolates from farmed Norwegian cod (strain NVI 5482) and Icelandic lump sucker (strain F57). The more distantly related strains form clade 1, which contains isolates from Canadian (strain Vvi-7 and Vvi-11) and Icelandic (strain K58) farmed Atlantic salmon including Norwegian farmed trout (strain NVI 4917 and NVI 5450). While the phylogenetic tree built from SNPs in the core region of the genomes and hence represents the vertical evolution, the gene content tree counts presence and absence of genes in isolates relative to each other and hence represents the horizontal evolution of the isolates. To test whether the uptake and loss of MGEs was the main driver of the *M. viscosa* evolution, the congruency between SNP phylogeny and the gene content tree was tested. The comparison revealed that the topology of the trees was similar and that the majority of clades are congruent in both trees resulting in a Robinson Fould Distance of 0.30 [44]. This gives further support to the relationships among the divergent *M. viscosa* lineages. Only NVI 5450 had a different placement. The comparative analysis between typical and variant *M. viscosa* revealed 231 genes shared between typical *M. viscosa* but which were not present in other variant *M. viscosa*. Of the 231 genes, 126 are annotated as putative uncharacterized proteins. A high number of the remaining predicted genes are homologues to predicted genes in other *Moritella* and *Vibrio* spp.

### Plasmid-like elements in *M. viscosa* and their putative encoding genes

From the comparative genomic study, we observed putative plasmid-like elements. We describe here the elements with complimentary sequences to spacers present in the CRISPR loci. One, which is present in MT 2528 (MT2528_3989 to MT2528_3955) and K56 (K56_4570 to K56_4597) is termed plasmid A. The analysis of plasmid A revealed nine genes encoding homologues to Trb proteins indicative of a P-type conjugation system. Also a putative type II-like secretion system (T2SS) protein, a hypothetical type IV (T4) pilin and a number of uncharacterized proteins were predicted indicative that the cluster encodes a T2SS or T4 pilus like transport system. The top ranking model for K56_4594 (and equivalent MT2528_4809) predicted by Phyre2 [43] is the *Vibrio cholerae* VesB protease (PDB template c4lk4A, model not shown). 80% of the sequence (residues 23–317) was modelled with 100.0% confidence with an N-terminal signal peptide and a C-terminal domain similar to an immunoglobulin (Ig) fold with a membrane spanning helix at the C-terminal end.

The putative plasmid B element in NVI 5482 (NVI5482_4403 to NVI5482_4431) contains genes encoding homologues to Tra proteins indicative of an F-type conjugation system. Blast searches of amino acid sequences to modules of the plasmid show highest identity to other marine bacteria such as *Aliivibrio salmonicida*, *Shewanella baltica*, *Aeromonas salmonicida* and *Photobacterium* sp.

The plasmid C element in MT 2528, NVI 5482, F57 and K56 (K56_4540 to K56_4568) is intriguing and may be remnants from a larger plasmid-like element as annotation reveals hallmarks (results not shown) for linear plasmid-like prophages reported from other Gram-negative marine bacteria [45]. The *repA* adjacent sequence stretches are not similar between Mt 2528, NVI 5482 and F57, and it is possible that the assortment of genes originates from sequence assembly difficulties. Most CDSs are annotated as uncharacterized proteins but several genes encode transposases, integrases, DNA modifying proteins, and phage related proteins.

In addition, all the predicted plasmid-like elements provided Pfam predicted relaxases using the Pfam-web tool [46].

### Prophages in *M. viscosa* genomes

The PHAST predicted prophages were separated into lineages according to their predicted similarity to known prophages and by their conserved synteny of the genomic structure. Predicted prophages (Additional file 4: Figure S1) found in two or more of the twelve sequenced *M. viscosa* genomes are presented as prophage 1–9. Prophage distribution between *M. viscosa* strains was then resolved by manually allocating similar structured prophages to one of the nine prophage types as shown in Fig. 2bc. The topology of the SNP and gene content trees is congruent, and comparing the prophage presence to the tree topology shows that the distribution of these prophages make patterns that support an evolutionary relatedness in the *M. viscosa* genomes. Only a small number of proteins can be related to known functions (Additional file 4: Figure S1). Genes for which function can be predicted are putative integrases, terminases and phage-structural proteins. Six of the prophage types contain integrases. A phylogenetic analysis based on the amino acid sequence of these integrases cluster in accordance to the predicted prophages supporting the allocation of these prophages to the correct prophage-type (Additional file 4: Figure S2). In addition to phage protein orthologs, attL and attR sites for site-specific integration into the genome and integrases were detected (Additional file 4: Table S3). The attachment sites are identical to the specific integrases that are phylogenetically related. All of the predicted attachment sites are repeatedly found throughout the genomes (results not shown).

### The CRISPR-Cas system in *M. viscosa*

**Fig. 3:**
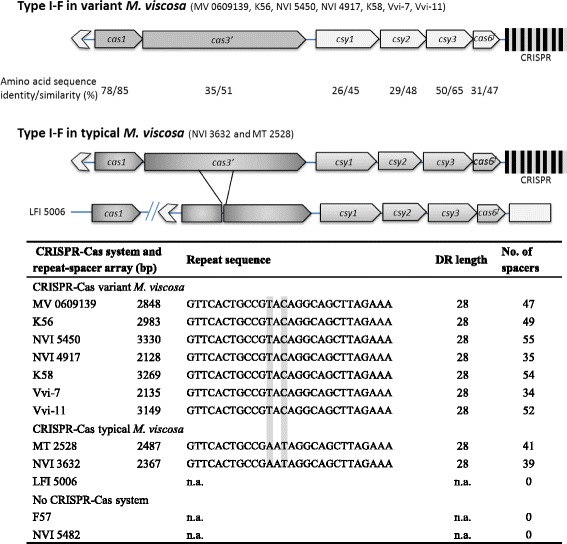
The CRISPR-Cas system in *Moritella viscosa*. Genetic composition of the CRISPR-Cas subtype I-F system in variant (upper) and typical *M. viscosa,* which also illustrates the truncated CRISPR-cas system in *M. viscosa* LFI 5006. The exactly matched amino acids (% sequence identity) and calculated (%) amino acid sequence similarity between typical and variant sequence pairs are shown between the illustrations. The below table shows the characteristics of CRISPR spacer-arrays in *M. viscosa.* Grey shaded letters mark deviations in nucleotides

Two distinct variants of the CRISPR-Cas system with amino acid sequence score alignments ranging between 26-78% identity were identified in *M. viscosa* (Fig. 3). They are divided between the variant *M. viscosa* clade 1 and clade 2, and typical *M. viscosa* (Fig. 2a). Both systems are classified by the system of Makarova et al. 2011 to belong to subtype I-F and include six genes (Fig. 3). Nucleotide alignments of the *cas* and *csy* genes show 100% nucleotide identity between all typical *M. viscosa* isolates harbouring these genes (except the truncated version of *cas3*’ in LFI 5006). The CRISPR-Cas genes were also conserved within variant *M. viscosa* (>99.9% identity) with the presence of a single conserved SNP. The *cas* operon encodes Cas1, Cas3’, and the subtype specific proteins Csy1, Csy2, Csy3 and Cas6^f^ (formerly Csy4) followed by a repeat-spacer array with the number of spacer per strain ranged from 0 to 55 (Fig. 3). However, LFI 5006 possesses a truncated *cas3*’ in addition to a dispersed *cas1* gene. These genes are required for integrating new spacer sequences [47], and could explain the lack of a predicted repeat-spacer array in this strain. The partly palindromic repeat sequences differ by two nucleotide substitutions between typical and variant *M. viscosa* CRISPR-arrays (Fig. 3). The closest experimentally validated CRISPR-Cas system to variant *M. viscosa* predicted by BLAST searches is the CRISPR-Cas system of *Pectobacterium atrosepticum* [48] (Additional file 4: Table S4). No CRISPR-Cas system could be identified in *M. viscosa* F57 and NVI 5482 (variant clade 3) using the same method. Further support for this observation was found using the flanking regions of the CRISPR-Cas. Downstream of the operons harboured an ABC transporter and a cold-shock DNA-binding domain family protein genes. Nucleotidyltransferase or a ferrous iron transport protein gene was identified upstream. Using these genes, the same regions were identified in F57 and NVI 5482 without signs of any CRISPR-Cas.

### Protospacer sequences are shared in related *M. viscosa*

**Fig. 4:**
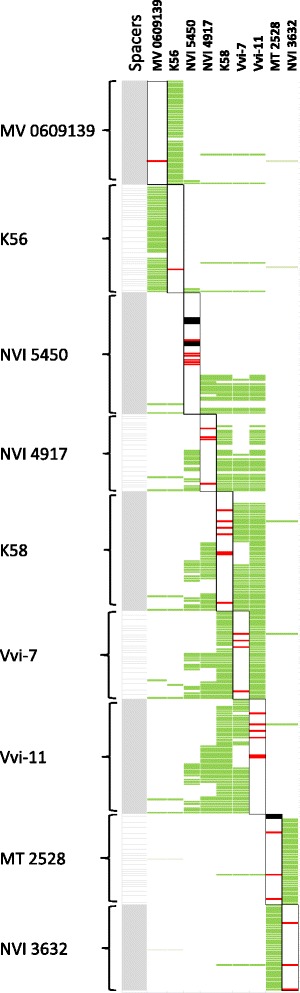
CRISPR profiles in nine *Moritella viscosa* strains indicating matches between spacers or other genomic elements in *M. viscosa*. Each spacer inn all *M. viscosa* strains are presented in numerical order (latest acquired is first) in the first row. Spacer ID’s in Additional file 3: Data S1 are named by its bacterial affiliation and numbered progressively where the highest number designates the last obtained spacer. Percentage and the number of identical spacers shared between *M. viscosa* strains are shown in detail in Table 1. Green spacers indicate identical spacers found in two or more *M. viscosa* strains. Light green indicate spacers with one mismatch to spacers in other *M. viscosa* strains. Black spacers indicate spacers found in specific *M. viscosa* spacer-arrays that are identical to prophage genes identified in other *M. viscosa* strains. Red spacers indicate spacers found in specific *M. viscosa* spacer-arrays that are identical to plasmid genes identified in other *M. viscosa* strains

In total, 412 spacers were identified among the nine CRISPR carrying *M. viscosa* (Fig. 4 and Table 1). Searches against the GenBank-Phage and RefSeq-Plasmid databases revealed only two spacer matches (defined as ≤4 SNPs = 28/32 nucleotides). Spacer 4919r6 matched *Gluconobacter oxydans* 621H plasmid pGOX1, while spacer 5450r53 matched an *Oenococcus* phage sequence. Comparing the spacers within our isolate collection identified 57 unique spacers mostly at the leader proximal end, which implies that they are the most recent spacers in terms of acquisition. The structure and similarity of the repeat-spacer arrays show a high heterogeneity of spacer content among *M. viscosa.* Overall, three main genotypes of spacer-sets could be assigned to variant clade 1, variant clade 2 and typical *M. viscosa* isolates (Fig. 4, Table 1), congruent to strain evolutionary relationships. The commonality between spacer-arrays in typical *M. viscosa* strains reflects the phylogenetic clustering of typical *M. viscosa*. The more distantly related isolates of clade 2 contain a different spacer-array set, which is conserved in synteny among clade 2 strains. The spacer-arrays in variant clade 1 *M. viscosa* is further comparable to strain evolutionary distance. Meaning that closely related isolates, e.g. K58, Vvi-7 and Vvi-11, are also displaying more similar repeat-spacer arrays, which become more variable with phylogenetic distance (compared to NVI 4917, or even further to NVI 5450). Trout isolates of clade 1 show a spacer-array pattern of similar origin but with a higher diversity in the more recent acquired spacers compared to Atlantic salmon isolates. The anchor spacer is the oldest spacer in terms of acquisition. This spacer is attained identical in all variant clade 1 and clade 2 strains. Strain K56 and MV 0609139 (clade 2) spacer-arrays are very similar in structure to each other and two spacers are identical to spacers in the arrays of the remaining variant strains. One spacer in typical *M. viscosa* is found in variant spacer-arrays.

**Table 1:**
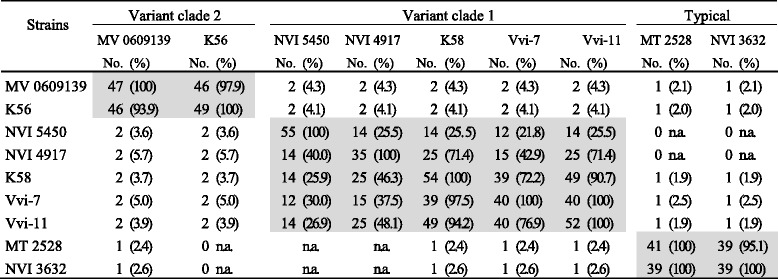
Identical spacers shared between *Moritella viscosa* strains

Grey shading indicates the three CRISPR-array genotypes in *M. viscosa*. Quantities of identical spacers are displayed as the specific numbers with the percentage of the total spacer array in brackets

### Protospacer containing prophage and plasmid-like CDSs in *M. viscosa*

BLAST searches of all *M. viscosa* spacers against the *M. viscosa* genomes revealed complimentary sequences (protospacers) that were part of prophage related genes (Additional file 4: Figure S3). MT 2528 spacers 2528r41 and 2528r40 are identical in sequence to prophage 1 and prophage 2, respectively. Concurrently, prophage 1 and 2 are predicted in the typical *M. viscosa* for both NVI 3632 and LFI 5006, except MT 2528 (Fig. 2). Spacer 2528r40 is also similar with one mismatch to prophage 7. NVI 5450 spacers 5450r24 and 5450r25 are identical in sequences to two genes predicted within prophage 4, while spacers 5450r13, 5450r14 and 5450r15 are similar to three genes in prophage 5.

Identical protospacers were also identified in the *M. viscosa* genomes to plasmid related genes (Additional file 5). Thirty-three spacers matched sequences within three putative *M. viscosa* plasmid designated A, B and C. Spacers within *M. viscosa* strains from variant clade 1 and clade 2 in addition to typical *M. viscosa* matched to plasmid A. Strains from variant clade 1 had spacers against plasmid B and plasmid C. Genes in plasmid A and B that contain one or more protospacers are predicted with functions that are essential to conjugative transfer. In plasmid A, *trbC*, *trbJ*, *trbL* and *traG* are targeted in addition to an uncharacterized protein gene (K56_4586 and MT2528_4007) and a putative serine protease (K56_4594 and MT2528_4809). In plasmid B, the conjugative transfer genes *traN*, *traE* and the *repA* gene encoding the putative replication protein, are targeted. Spacer K56r10 and Vvi-11r8 identical to protospacer sequence in plasmid B *repA* are also similar with three mismatches to the plasmid C *repA*, which could be caused by the sequence similarity. It is noteworthy that spacers in variant clade 1 strains repeatedly match the plasmid C *repA* gene. Both consecutive spacers, as well as spacers that are acquired at different time points (other spacers are between them) are observed.

## Discussion

This study presents the first comparative genome analysis of *M. viscosa*. Analyses of the genome plasticity among strains revealed that vertical and horizontal evolution relationships are concurrent to each other. By predicting the function of accessory and unique genes among *M. viscosa,* it was revealed that many of the genes resulted from predicted MGEs such as prophages and plasmids. We further used genome structure characteristics to investigate if *M. viscosa* has mechanisms for acquired immunity against MGEs. Two subtypes of the CRISPR-Cas I-F system were identified. The distribution of these systems and the spacer-array variants correlate with the phylogenetic lineage pattern. The whole-genome phylogenies indicate four *M. viscosa* lineages expanding the previously suggested classification of typical and variant *M. viscosa* [5, 8], which might suggest that sublineage definition among *M. viscosa* needs revision. Spacer-arrays within each lineage are conserved in synteny. In contrast, little commonality is observed between each lineage. That spacer composition can be linked to *M. viscosa* population structure and evolutionary relationships is similar to other bacteria [49]. CRISPR typing can provide tracking and subtyping of pathogenic strains [18–20, 50, 51]. Strain typing and tracking of *M. viscosa* could potentially enhance our understanding of the ecological context of infectious winter-ulcer disease. However, a broader range and number of isolates are needed to establish such a method as there is no evident phylogenetic or genotypic pattern that associate *M. viscosa* subgroups to geographic distribution or with host type from the isolates used in this study.

That only two *M. viscosa* CRISPR-spacers matched to protospacer sequences of known plasmids and phages could be a result of the expected large variety of MGEs present in marine environments. Functionality of CRISPR-Cas where spacer sequences provide prophage resistance [52] and limit plasmid transfer [53] in *M. viscosa* was indicated by the correlation between the CRISPR-spacer content and the distribution of the matching MGE. That MGE-matching CRISPR-spacers are excluded is observed in MT 2528 where the two unique and most recent acquired spacers match prophages present in typical *M. viscosa*, except MT 2528. Similarly, CRISPR-spacers in NVI 5450 match to prophages present in other *M. viscosa* strains, but which are absent in NVI 5450. Supporting this model, plasmid B is absent from genomes containing matching CRISPR-spacers, but is present in NVI 5482 without any matching CRISPR-spacers. Plasmid C is similarly predicted in genomes lacking matching CRISPR-spacers.

Divergence to this model is observed between NVI 3632 and MT 2528 that both have two CRISPR-spacers directed at plasmid A. NVI 3632 is not predicted with plasmid A but MT 2528 harbours plasmid A. The CRISPR-arrays are identical except for the two most recent prophage spacers in MT 2528, which suggests a functional CRISPR-Cas in MT 2528. The possibility of CRISPR autoimmunity is rejected, as plasmid A spacers do not match any of the CRISPR-Cas gene sequences, which in addition are identical at the nucleic acid level ruling out any recent mutational effect causing inefficient or defective CRISPR-Cas system in MT 2528. The reason is not known but the escape from the CRISPR-Cas system could be caused by mutations in other sequence motifs, which is known to avoid recognition [48]. It is interesting to note that these spacers are acquired at two different time points with 30 in-between-spacers suggesting multiple interactions with this plasmid-type. Strain K56 harbor a CRISPR-spacer with 1 bp spacer-mismatch to plasmid A, which could explain how this plasmid evade CRISPR-Cas immunity in this strain. Mutations in the targeted MGE can lead to repetitive acquisition or incorporating of new spacers to the CRISPR-array that again increase resistance against the invading MGE [54]. The repetitive acquisition of spacers, in addition to spacers that show mismatches to essential genes within MGEs predicted in this study, suggests reoccurring encounters or interaction with variants of these MGEs at previous time points as described in other marine bacteria [49]. The existence of a co-evolutionary “arms race” where CRISPR immunity drives MGE evolution [16] may also occur between *M. viscosa* CRISPR-Cas and their targets.

The CRISPR-Cas targeted prophage genes in *M. viscosa* are essential for genome integration and to a prophage life cycle. Targeted plasmid genes are essential for replication or conjugation. Essential genes are often more conserved in sequence conservation, meaning that targeting these genes would confer a more efficient immunity over an extended period. It is noteworthy that *repA* in plasmid C (but also plasmid B) is repetitively targeted by the CRISPR-Cas system in variant clade 1. This might be due to spacer acquisition preferences as CRISPR-Cas target plasmids in preferentially regions [55]. Alternatively, genetic elements could acquire escape mutations or genetic shuffling that elude the CRISPR-Cas immunity [56] and adapt to infect their environment preferential host type [57] being able to repeatedly infect the host as observed in the distance between acquired spacers in the CRISPR-array. In the *Escherichia coli* plasmid prophage N15, *repA* is the only gene necessary for replication [58]. Targeting this gene will provide defence against all variants of MGEs containing this or related *repA* with matching protospacers and could suggest that *M. viscosa* CRISPR-Cas also targets MGEs in a meticulous manner.

CRISPR-Cas mediated immunity can provide bacteria an advantage in the presence of a lytic phage [59]. It is shown that temperature may induce bacterial stress responses that activate the lysogenic switch of prophages [60]. Although, no lysis module was predicted in *M. viscosa* prophages, it cannot be excluded that prophages may play a role in the lytic switch of *M. viscosa* observed above 10 °C [61] and be a situation where CRISPR-Cas mediated immunity provide an advantage in *M. viscosa*. Targeting of conjugative plasmids is likely dependent on if plasmid genes may become a burden in particular environments or not [62, 63]. Spacers matching to conjugative transfer genes in *M. viscosa* could suggest that some conjugative plasmids impose an unwanted burden in *M. viscosa.* Targeting of unessential plasmid genes indicates additional specific genetic elements unwanted in *M. viscosa* such as the plasmid A encoding T2SS / T4 pilus-like transport system. It is unknown if the system could affect the genomic T2SS but the complex is likely driving the translocation of the predicted trypsin-like serine protease that share structural similarities to VesB, a T2SS exoprotein in *V. cholerae* [64]. Transportation is supported by the predicted N-terminal signal peptide similar to other proteases that enters the periplasm via the Sec pathway before T2SS [65]. The protease has similar to VesB a predicted Ig-fold of unknown function [66]. Ig-like domains are found in several types of cell surface proteins involved in substrate specificity or surface recognition [67]. Expression of plasmid A genes could alter host cell adhesion and invasion properties of *M. viscosa* or alternatively result in autolysis similar to the T2SS translocated serine protease in *Vibrio vulnificus* [68].

The high population density and eutrophic environment in fish farming could have selected for and facilitated the rapid strain flow of host specific typical *M. viscosa* [8] in Norwegian Atlantic salmon aquaculture compared to the more diversification of the pathogen in other fish species and geographical areas [5]. If assumed that CRISPR-arrays are an indirect reflection of the environment, i.e. it reflects the type of MGEs encountered in the environment occupied by the bacteria, it will indicate that the different sublineages originate from different environments. However, a variety of mechanisms unrelated to CRISPR-Cas conferred immunity could affect the sensitivity to MGEs [69]. It is further postulated that CRISPR-Cas systems are lost when they confer immunity to acquired beneficial genes, and subsequently regained in environments where protection against MGEs again increase fitness [70]. The CRISPR-Cas of variant clade 3 *M. viscosa* could have similarly been lost during clade specific evolution. This lineage is the closest in relationship to typical *M. viscosa*, which has a separate CRISPR-Cas system that could have been gained in response to a different environment. Although the CRISPR genotypes are distinct, they are all found in isolates from salmonids. This could relate to a relatively isolated niche in which these strains are isolated and could indicate that CRISPR-Cas inferred immunity has a positive consequence in the eutrophic environment of fish farming.

## Conclusions

From the comparative genome analysis in this study, we describe how the genome plasticity and relationships among *M. viscosa* is reflected by MGEs. The correlation between CRISPR-spacers that matches protospacers suggests that CRISPR-Cas confer adaptive immunity against MGEs in *M. viscosa*, and is a counter-strategy acquired in multiple events. Moreover, our findings suggest that CRISPR-Cas and their spacer-array contents originating from foreign DNA correlate with the evolutionary relationships among *M. viscosa* that could provide a new tool for evaluating diversity and strain tracking of *M. viscosa.*

## Additional files

Additional file 1: Table S1. *Moritella viscosa* isolates genome sequenced in this study. (DOCX 31 kb)

Additional file 2: Table S2A. Summary of genomic statistics of *M. viscosa.* **Table S2B.** Sequence statistics. **Table S2C.** Orthologue statistics. **Table S2D** Comparative genome analysis. (XLSX 1103 kb)

Additional file 3: Data S1. *M. viscosa* CRISPR spacers. (TXT 17 kb)

Additional file 4: Figure S1. Prophages in *M. viscosa*, **Figure S2.** Prophage integrase phylogeny, **Table S3.** Prophage att sites, **Table S4.** Homology analysis of CRISPR-Cas systems identified in *Moritella viscosa,* **Figure S3.** Prophage protospacers. (DOCX 442 kb)

Additional file 5: Table S5A. *M. viscosa* spacers matching prophage-like elements. **Table S5B.** *M. viscosa* spacers matching plasmid-like elements. (XLSX 18 kb)

## Abbreviations

BLAST: Basic local alignment search tool
CDS: Coding sequence
CRISPR-Cas: Clustered regularly interspaced short palindromic repeats and associated Cas proteins
HGT: Horizontal gene transfer
MGE: Mobile genetic element
PHAST: Phage search tool
SNP: Single-nucleotide polymorphism
T2SS: Type II secretion system
