# Supplementary material for: Pan genome and CRISPR analyses of the bacterial fish pathogen Moritella viscosa
Source: BMC Genomics. 2017 Apr 20;18:313. doi: 10.1186/s12864-017-3693-7 (PMC5399434; doi:10.1186/s12864-017-3693-7)
Supplement: Supplementary file 1 — Moritella viscosa isolates genome sequenced in this study. (DOCX 31 kb) [file 12864_2017_3693_MOESM1_ESM.docx]

Table S1 *Moritella viscosa* isolates genome sequenced in this study

| *Moritella viscosa* | | | | | | Isolation origin | ECP | Atlantic salmon challenge | | |
| --- | --- | --- | --- | --- | --- | --- | --- | --- | --- | --- |
|  | |  | | [year] | | | Lethality^a^ | Method^b^  LD_50_ Ref.^c^ | | |
| **Hosted in *S. salar*** | | | | | | |  |  |  |  |
|  | LFI 5006* | | Norway [2002] | | | | Yes | bath | n.d. | [71] |
|  | NVI 3632 | | Norway [1996] | | | | n.d. | bath | <1.2 x 10^6^ | [8] |
|  | MV 0609139 | | | | Norway [2006] | | n.d. | bath | <1 x 10^6^ ml^-1^ | [23] |
|  | K58 | | Iceland [1992] | | | | Yes | im  ip | <1.5 x 10^1^  2 x 10^5^ | [3]  [21] |
|  | K56 | | Iceland [1994] | | | | Yes | im | <1.1 x 10^2^ | [1] |
|  | MT 2528 | | Scotland [2001] | | | | Yes | ip | 2 x 10^4^ | [21] |
|  | Vvi-11 | | Canada [2005] | | | | No | ip | avirulent | [21] |
|  | Vvi-7 | | Canada [2005] | | | | No | ip | avirulent | [21] |
| **Hosted in *O. mykiss*** | | | | | | |  |  |  |  |
|  | NVI 4917 | | | Norway [2004] | | | Yes | ip | 7 x 10^5^ | [21] |
|  | NVI 5450 | | | Norway [2006] | | | Yes | bath | >5 x 10^5^ | [8] |
| **Hosted in *G. morhua*** | | | | | | |  |  |  |  |
|  | NVI 5482 | | | Norway [2006] | | | Yes | ip | 2 x 10^5^ | [21] |
| **Hosted in *C. lumpus*** | | | | | | |  |  |  |  |
|  | F57 | | | Iceland [-] | | | Yes | im  ip | 1.7 x 10^6^  >2 x 10^6^ | [1]  [21] |

^a^Reference [21]. ^b^ip, intraperitoneal; im, intramuscular; n.d., not determined. ^c^References as in main text except [71. Mikkelsen H, Eggset G: Challenge of Atlantic salmon with *Vibrio viscosus, V. wodanis* and a combination of both species. EAFP Conference Sept 19-24, 1999 Rhodos, Greece 1999]. *LFI 5006 is an isolate from dead Atlantic salmon (2002) experimentally challenged with LFI 5000, which was originally isolated from Atlantic salmon suffering from a natural occurring outbreak in 1997.
