# Supplementary material for: Pan genome and CRISPR analyses of the bacterial fish pathogen Moritella viscosa
Source: BMC Genomics. 2017 Apr 20;18:313. doi: 10.1186/s12864-017-3693-7 (PMC5399434; doi:10.1186/s12864-017-3693-7)
Supplement: Supplementary file 4 — Prophages in M. viscosa, Figure S2. Prophage integrase phylogeny, Table S3. Prophage att sites, Table S4. Homology analysis of CRISPR-Cas systems identified in Moritella viscosa, Figure S3. Prophage protospacers. (DOCX 442 kb) [file 12864_2017_3693_MOESM4_ESM.docx]

Figure S1 Prophage structures


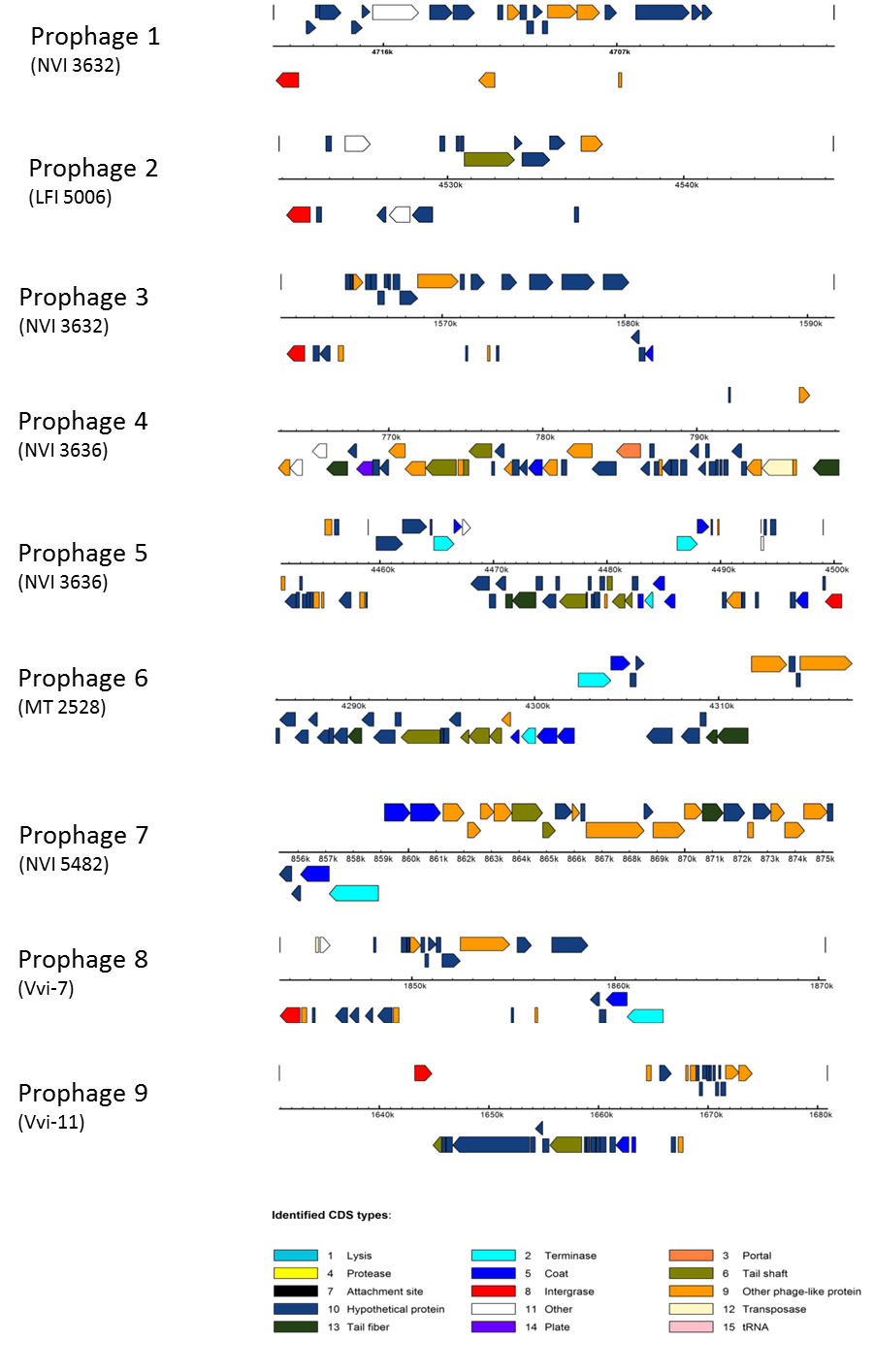


Figure S1 The detailed structures of putative prophages found in two or more *Moritella viscosa* genomes. The identification has been performed using PHAST software. Schematic diagrams are shown for predicted prophage 1 – 9 using the genome location and prophage region denoted by the *M. viscosa* strain in brackets. The functional classification of the predicted prophage proteins are indicated by color codes. Black thin bars in prophages 1, 2, 3, 5, 8 and 9 are putative internal attL and attR sites for site-specific recombination.

Figure S2 Prophage integrase phylogeny


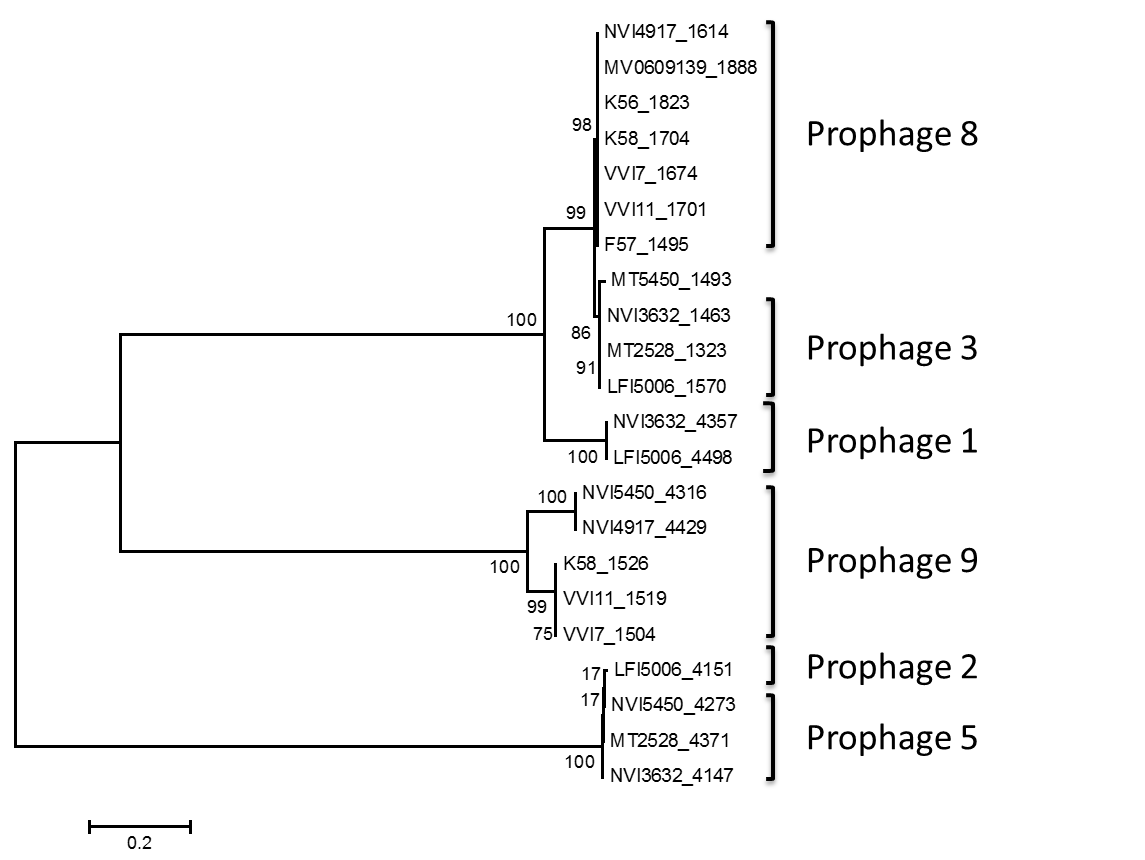


Figure S2 The phylogenetic relationships based on the alignment of the amino acid sequence of the prophage integrases extracted from the genomes. Sequences were aligned using the ClustalW algorithm in BioEdit and their relationships inferred by using the Neighbour-Joining (NJ) (poisson correction) method with pairwise deletion and 1000 bootstrap trials in MEGA5.

Table S3 Prophage att sites

| **Prophages** | **Integrase gene** | **Predicted prophage region (kbp)** | **Putative attachment site** | **Genomic position att L** | **Genomic position att R** |
| --- | --- | --- | --- | --- | --- |
| Prophage 1 | NVI3632_4357 | 24 | aaaaggctccctaaggag | 47221732..4721749 | ND |
|  | LFI5006_4498 | ND | aaaaggctccctaaggag | 4903921..4903938 | ND |
|  |  |  |  |  |  |
| Prophage 2 | LFI5006_4151 | 23 | tatttattttaa | 4522856..4522867 | 4546332..4546343 |
|  | NVI3632_ND | ND |  | ND | ND |
|  |  |  |  |  |  |
| Prophage 3 | NVI3632_1463 | 73 | gttaacagctgttt | 1561370..1561383 | 1634369..1634382 |
|  | MT2528_1323 | 69 | gttaacagctgttt | 1408875..1408889 | 1478035..1478049 |
|  | LFI5006_1570 | 27 | gttaacagctgttt | 1687009..1687022 | 1713785..1713798 |
|  | NVI5450_1493 | 34 | gttaacagctgttt | 1611763..1611775 | 1645562..1645574 |
|  |  |  |  |  |  |
| Prophage 5 | NVI3632_4147 | 40 | taaataaaaaaat | 4458963..4458975 | 4499038..4499050 |
|  | MT2528_4371 | ND | taaataaaaaaat | 4739636..4739649 | ND |
|  | NVI5450_4273 | ND | taaataaaaaaat | 4662726..4662737 | ND |
|  |  |  |  |  |  |
| Prophage 8 | MV0609139_1888 | 35 | tttaaaatataa | 2011103..2011114 | 2046284..2046295 |
|  | F57_1495 | 35 | tttaaaatataa | 1600199..1600211 | 1644385..1644396 |
|  | VVI11_1701 | 24 | tttaaaatataa | 1852816..1852827 | 1876423..1876434 |
|  | VVI7_1674 | 27 | tttaaaatataa | 1843506..1843517 | 1870331..1870342 |
|  | K58_1708 | 22 | tttaaaatataa | 1863887..1863898 | 1885951..1885962 |
|  | NVI4917_1614 | 42 | tttaaaatataa | 1776182..1776193 | 1818660..1818672 |
|  | K56_1823 | 20 | tttaaaatataa | 1934522..1934533 | 1954176..1954187 |
|  |  |  |  |  |  |
| Prophage 9 | NVI5450_4316 | 52 | ccaaaaaaaaca | 4702016..47029 | 4753830..4753841 |
|  | NVI4917_4429 | 48 | cataaaaaaaca | 4821704..4821715 | 4869656..4869667 |
|  | K58_1526 | 40 | gcaataccattacc | 1646292..1646307 | 1696154..1686167 |
|  | VVI11_1519 | 50 | gcaataccattacc | 1630867..1630880 | 1680891..1680904 |
|  | VVI7_1504 | 59 | gcaataccattacc | 1621089..1621102 | 1679886..1679898 |
|  |  |  |  |  |  |

ND = not determined as identical attachment sites were not confirmed at both ends of the prophage

| CRISPR associated  genes |  | Homology to ORFs of other species | | | | | | | | |  | Variant vs typical  *M. viscosa* |
| --- | --- | --- | --- | --- | --- | --- | --- | --- | --- | --- | --- | --- |
|  |  | Variant *M. viscosa* K58 | | | |  | Typical *M. viscosa* 3632 | | | |  |  |
|  |  | Predicted no. of aa | Homologous locus | Species | % identity/  similarity |  | Predicted no. of aa | Homologous locus | Species | % identity/  similarity |  | % identity/  similarity |
| Cas1 |  | 331 | [Q02ML7](http://www.uniprot.org/uniprot/Q02ML7) | *Pseudomonas aeruginosa* | 59/73 |  | 335 | [Q02ML7](http://www.uniprot.org/uniprot/Q02ML7) | *Pseudomonas aeruginosa* | 57/72 |  | 78/85 |
| Cas3 |  | 1131 | [Q6D0W9](http://www.uniprot.org/uniprot/Q6D0W9) | *Pectobacterium atrosepticum* | 47/62 |  | 1118 | [Q02ML8](http://www.uniprot.org/uniprot/Q02ML8) | *Pseudomonas aeruginosa* | 38/55 |  | 35/51 |
| Csy1 |  | 435 | [Q6D0W8](http://www.uniprot.org/uniprot/Q6D0W8) | *Pectobacterium atrosepticum* | 40/59 |  | 419 | [A1SUQ0](http://www.uniprot.org/uniprot/A1SUQ0)* | *Psychromonas ingrahamii* | 68/83 |  | 26/45 |
| Csy2 |  | 321 | [Q6D0W7](http://www.uniprot.org/uniprot/Q6D0W7) | *Pectobacterium atrosepticum* | 44/62 |  | 306 | [A1SUQ1](http://www.uniprot.org/uniprot/A1SUQ1)* | *Psychromonas ingrahamii* | 68/84 |  | 29/48 |
| Csy3 |  | 326 | [Q6D0W6](http://www.uniprot.org/uniprot/Q6D0W6) | *Pectobacterium atrosepticum* | 62/74 |  | 341 | [Q6D0W6](http://www.uniprot.org/uniprot/Q6D0W6) | *Pectobacterium atrosepticum* | 52/67 |  | 50/65 |
| Cas6^f^ |  | 183 | [Q6D0W5](http://www.uniprot.org/uniprot/Q6D0W5) | *Pectobacterium atrosepticum* | 51/66 |  | 191 | [Q1RE32](http://www.uniprot.org/uniprot/Q1RE32) | *Escherichia coli* | 36/55 |  | 31/47 |

Table S4 Homology analysis of CRISPR-Cas systems identified in *Moritella viscosa*

*un-characterized

Figure S3 Prophage protospacers **
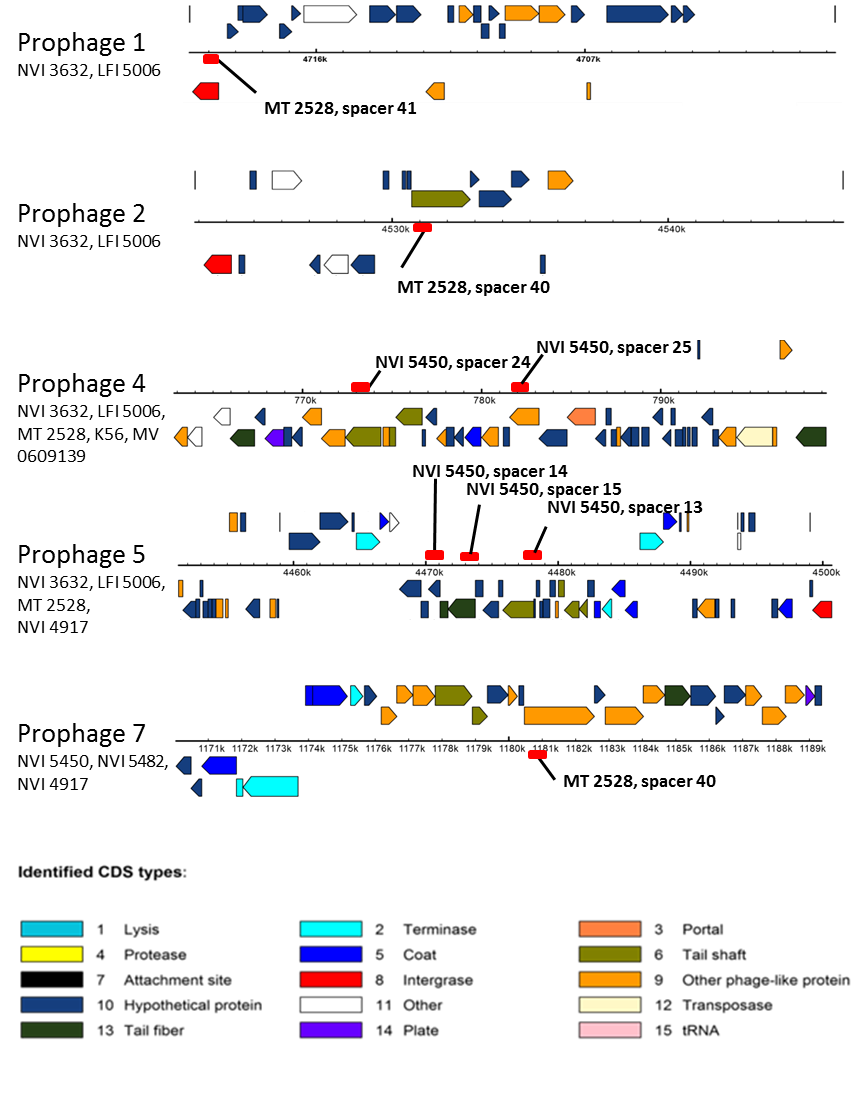
**

Figure S3 The prophage identification has been performed using PHAST software. Schematic diagrams are shown for predicted prophage 1, 2, 4, 5 and 7 with protospacers. Prophage distribution is denoted by the *M. viscosa* strains. The functional classification of the predicted prophage proteins are indicated by colour codes. Black thin bars in prophages 1, 2 and 5 are putative internal attL and attR sites for site-specific recombination. Horizontal red bars indicate the location of the protospacer where *M. viscosa* strains with corresponding matching CRISPR-spacer are denoted.
